# Chemical and Electrochemical Recycling of End-Use Poly(ethylene terephthalate) (PET) Plastics in Batch, Microwave and Electrochemical Reactors

**DOI:** 10.3390/molecules25122742

**Published:** 2020-06-13

**Authors:** Tessa H. T. Myren, Taylor A. Stinson, Zachary J. Mast, Chloe G. Huntzinger, Oana R. Luca

**Affiliations:** Department of Chemistry and Biochemistry, 215 UCB., University of Colorado Boulder, Boulder, CO 80300, USA; Tessa.myren@colorado.edu (T.H.T.M.); Taylor.stinson@colorado.edu (T.A.S.); zachary.mast@colorado.edu (Z.J.M.); chloe.huntzinger@colorado.edu (C.G.H.)

**Keywords:** polymer recycling, microwave, electrochemical reactor, quaternary recycling, depolymerization

## Abstract

This work describes new methods for the chemical recycling of end-use poly(ethylene terephthalate) (PET) in batch, microwave and electrochemical reactors. The reactions are based on basic hydrolysis of the ester moieties in the polymer framework and occur under mild reaction conditions with low-cost reagents. We report end-use PET depolymerization in refluxing methanol with added NaOH with 75% yield of terephthalic acid in batch after 12 h, while yields up to 65% can be observed after only 40 min under microwave irradiation at 85 °C. Using basic conditions produced in the electrochemical reduction of protic solvents, electrolytic experiments have been shown to produce 17% terephthalic acid after 1 h of electrolysis at −2.2 V vs. Ag/AgCl in 50% water/methanol mixtures with NaCl as a supporting electrolyte. The latter method avoids the use of caustic solutions containing high-concentration NaOH at the outset, thus proving the concept for a novel, environmentally benign method for the electrochemical recycling of end-use PET based on low-cost solvents (water and methanol) and reagents (NaCl and electricity).

## 1. Introduction

The rate at which municipal solid waste is generated and landfilled directly correlates to the use and disposal of plastic waste. An estimated 35.4 million tons of plastic were produced in 2017 in the United States. Of the plastic waste produced in the U.S., 5.01 million tons were poly(ethylene terephthalate) (PET) [1]. While PET is generally the most recovered plastic, the amount of waste PET that is recycled is still low, especially given the scale of use. This leads to an increased volume of landfills as discarded plastic is not biodegradable which contributes negatively to greenhouse gas emissions, and has detrimental effects on the world’s ecosystems, most specifically on marine life [2,3,4]. The most reported reason for lack of recycling is the inaccessibility of recycling resources.

**Figure 1:**
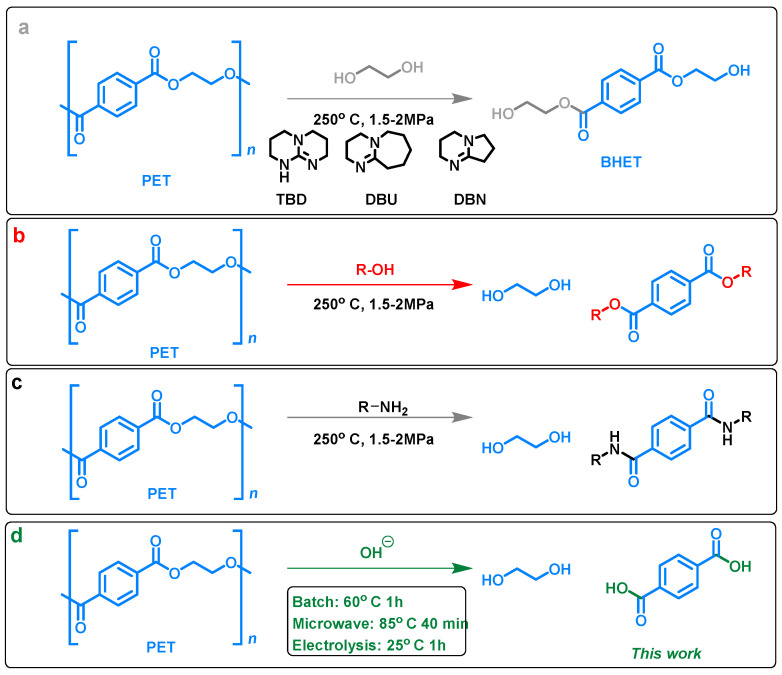
Chemical depolymerization methods for depolymerization of poly(ethylene terephthalate) (PET). (**a**) glycolysis (**b**) alcoholysis (**c**) aminolysis (**d**) chemical depolymerization methods in refluxing methanol and base in batch; at 85 °C in the microwave in 40 min; in an electrolysis experiment at room temperature.

A life cycle analysis [5] based on the twelve principles of green chemistry [6] by Landis and coworkers has highlighted the low biodegradability, high ecotoxicity and overall environmental impact of PET, despite extensive efforts in its recovery and recycling. While new biodegradable polymers become available [7], there is continued use and accumulation of PET that needs to be addressed. As such, improved methods for its reuse through recycling are sought. Recycling of PET can be divided into four main practices: primary, quaternary, mechanical, and chemical. Primary recycling involves recycling of PET before it reaches the hands of the consumer. This process re-extrudes the plastic material and requires that the plastic be clean and separated, such that primary recycling feedstock often comes directly from the plastic manufacturing plant [8]. The added need for separation significantly impacts the cost of the method. Mechanical recycling implements heat and mechanical force to break down the polymer and then incorporate it into another useable material without changing the chemical identity of the polymer [9]. Mechanical recycling is still a major recycling process, despite causing a decrease in molecular weights, due to its simplicity and low investment costs [9,10]. Solid-state polycondensation (SSP) is a physical modification process used in PET production to increase the molecular weight and quality of the polymer [11]. It is used following mechanical methods to make “bottle to bottle” recycling possible [12]. However, it is affected by contaminants and requires long reaction times and expensive control devices [10,13,14,15]. Quaternary recycling recovers energy from plastic by incineration [16]. This recycling method is process-intensive and produces greenhouse gases. While it is beneficial for multiple recycling methods to be implemented, the majority of the recycling performed on scale is not sustainable due to intensive use of resources and low-value recovery [16]. In contrast to primary, quaternary and mechanical processes, chemical recycling is a sustainable recycling option. Chemical recycling leads to the breakdown of the polymer esters into monomers or other smaller components, thus changing the chemical identity of the starting material, which can then be used to reform the polymer or produce valuable chemicals. The ultimate goal of chemical recycling—and the focus of this work—is to recover monomer materials to use as a feedstock for the production of new plastics and chemicals [9,16,17]. The Fraunhofer Institute in Germany is working on the upcycling of PET using chemical methods which do not require prior separation from other consumer waste [18]. The chemical recycling of PET can be further broken down into two main methods: glycolysis and hydrolysis (or alcoholysis). Glycolysis involves the insertion of ethylene glycol or its derivatives into the PET chain to produce a monomer of the PET polymer, bis-(hydroxyethyl) terephthalate, as shown in Figure 1a [19]. The conditions for this reaction often require high temperatures from 180 °C to 240 °C and high pressures [20]. Organic bases such as 1,5,7-triazabicyclo [4.4.0]dec-5-ene (TBD), 1,8-diazabicyclo[5.4.0]undec-7-ene (DBU), and 1,5-diazabicyclo[4.3.0]non-5-ene (DBN) can be used for the depolymerization of PET to monomers that can then be used for repolymerization [21]. Glycolysis is the oldest chemical PET recycling method and has been used commercially for more than 40 years [22]. Glycolysis uses TBD in an excess of ethylene glycol at 190 °C to convert PET to bis(2-hydroxyethyl)terephthalate (BHET), a monomer for polymerization of high-quality PET, in a 78% yield [23]. A related method, hydrolysis, entails the solvolytic cleavage of PET as shown in Figure 1b,c (alcoholysis and aminolysis respectively), and yields monomer forms ethylene glycol and derivatives of terephthalic acid: esters and amides, respectively. Like glycolysis, these reactions often require high temperatures upwards of 250 °C, and pressures between 1.5 and 2 MPa [16,19,20]. 

Hydrolysis can occur in neutral, acidic, or basic conditions. Under neutral conditions, steam can be used to depolymerize PET, although the reaction is slow in the absence of added inorganic salts or organic cosolvents [16]. The alkaline hydrolysis of PET is usually carried out in a concentrated solution of potassium hydroxide or sodium hydroxide. The four products of this reaction are ethylene glycol and the dipotassium or disodium terephthalate salt [20,24,25]. For the acidic hydrolysis of PET, concentrated sulfuric acid (>10 M), [25] nitric acid, and phosphoric acid [26] are used. Acid hydrolysis and alkaline hydrolysis are inherently corrosive as high concentrations of acid or base can corrode the reaction vessels [20] and therefore shorter reaction times and lesser corrosive chemical recycling alternatives are necessary for the long term implementation of chemical recycling methods on scale.

## 2. Results and Discussion

Given the known recycling methods, the ubiquity of PET plastics, and the knowledge gaps identified in the field, this paper aims to provide additional chemical recycling alternatives for the recovery of monomeric materials from end-use PET. We first focused on the optimization of the basic hydrolysis of this end-use polymer. In purely aqueous conditions, we observed yields of terephthalic acid of up to 23% after 48 h at reflux (Entry 3, Table 1). Similar yields are obtained after only 4 h upon switching the solvent to methanol (Entry 1, Table 1). A 12 h reaction time produced 75% of the expected terephthalic acid (Entry 2, Table 1). We note that the maximum %yield of recovered terephthalic acid from end-use PET is affected by the presence of plasticizers and additives, including siloxanes which were detected using GC-EI. Please visit Section 4.5 for additional details. Tomari and coworkers report complete decomposition of PET plastic under similar conditions to occur in 7 h [24], with acceleration to 40 min of reaction time observed by adding a 10% volume of 1,4-dioxane. While their report is an important advance, ethereal solvents are peroxide formers [27] and pose a significant hazard when used industrially on large scale. They are most often stabilized by radical-inhibitors such as BHT. The presence of radical inhibitors introduces the need for additional separation steps and consequently raises costs [28].

Given the limitation of ethereal accelerants and advances in synthetic methodologies based on microwave reactors [29], combined with reports related to the recycling of PET plastics through microwave digestion in aqueous conditions with the use of phase-transfer catalysts [30], we proceeded to investigate the depolymerization of end-use PET in conditions similar to Table 1, Entry 2. In the microwave, temperatures above the solvent boiling points can be achieved, thus producing higher reaction rates and lowered reaction times. The results of our studies are summarized in Table 2.

In these experiments, under exclusively aqueous conditions, we did not observe any PET breakdown, even at a temperature of 170 °C (Table 2 Entry 1). Gratifyingly, in 13 min at 85 °C in methanol, we observed 55% terephthalic acid yield which increased to 65% with a longer reaction time of 40 min (Table 2 Entry 3). An increase in temperature to 130 °C did not produce a higher yield. We believe this is due to a combination of factors related to mass transport and stirring limitations in the reactor.

**Scheme 1:**
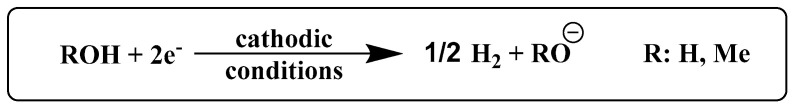
Electrochemical generation of base from protic media for the electrochemical depolymerization of PET plastic at neutral starting conditions.

Given our experience in the field of electrolytic hydrogen production [31,32,33] and reductive electrocatalysis [34,35], we investigated the electrochemical generation of the necessary basic conditions using current passage through a protic medium (Scheme 1). We hypothesized that this methodology would have a distinct advantage over the batch and microwave methods occurring at room temperature in neutral salted media, with the necessary base generated in situ (Scheme 1) at rates that one can control by dialing in the delivery of charge in coulombs/s. This electrochemical method avoids the use of corrosive solutions that may pose a significant limitation in the scaling of the chemical recycling process. The results of our studies are shown in Table 3.

Electrolysis in aqueous conditions at −2.2V vs. Ag/AgCl, (Table 3 Entry 3) resulted in low levels of breakdown of the PET plastic. This observation agrees with our prior results in the batch and microwave reactions. When the solvent is changed to 1:1 methanol/water, however, a one-hour electrolysis was able to produce a yield of 17% of terephthalic acid (Table 3 Entry 1). In addition, we also observe the formation of carbon dioxide in the headspace of our anode chamber, which likely correlates to the migration of the TPA across the frit of our electrochemical reactor and subsequent Kolbe decarboxylation [36,37]. This establishes a proof of concept for an electrochemical method for hybrid chemical and quaternary recycling, as an alternative to combustive methods.

## 3. Conclusions

In conclusion, we now report that the decomposition of PET plastic and recovery of terephthalic acid can be achieved with relatively mild conditions in alkaline methanol both in batch and in the microwave. Microwave reactions occurred with reaction times as short as 40 min at only 85 °C. In addition, we report that base generated in an electrochemical reaction in the presence of end use PET can cause the breakdown of the polymer at room temperature without the use of highly corrosive media. While the yields of the electrochemical reaction remain modest, they provide an important advance in the arena of sustainable chemical recycling on scale.

## 4. Materials and Methods 

### 4.1. General Methods

Materials, reagents, and solvents were obtained from commercial sources without further purification unless otherwise noted. ^1^H-NMR spectra for the characterization and yield analysis of depolymerization products were taken on a Bruker AV-III 300 MHz NMR Spectrometer (Billerica, MA, USA) at room temperature in DMSO-*d*_6_. Poly(ethylene terephthalate) (PET) was obtained from Sam’s Choice Purified Drinking Water bottles which were emptied, cleaned, and then sheered with scissors into smaller pieces. 

### 4.2. PET Depolymerization in Batch and Microwave

**Figure 2:**
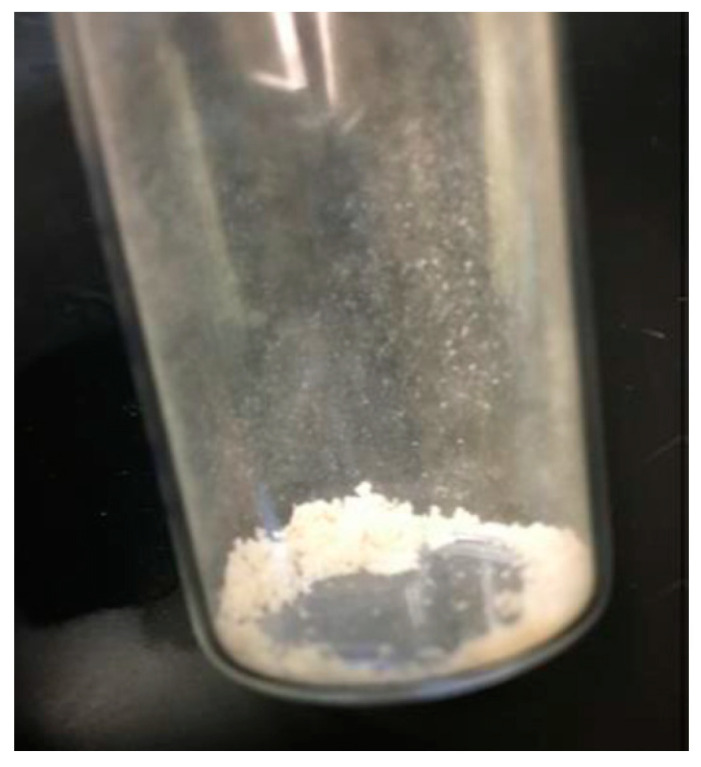
White powder collected after the workup.

PET was depolymerized in a 3.75 M solution of sodium hydroxide in methanol unless otherwise noted. The reaction solution was then placed in a round bottom flask and was dried on a rotary evaporator followed by high-vacuum to remove residual methanol. The resulting white solids consisted of sodium hydroxide and disodium terephthalate which were then re-dissolved in a minimal amount of water. For experiments run in water instead of methanol, this step was omitted. The flask was placed in a water ice bath with stirring. The solution was acidified with hydrochloric acid, and verified with pH paper, to obtain terephthalic acid (TPA). The flask was again placed on the rotary evaporator with a hot water bath followed by high-vacuum to dry. Ethyl acetate was added to the flask, heated to 60 °C, [38] then sonicated to dissolve the TPA and separate the depolymerization product from the sodium chloride salt. The TPA/ethyl acetate solution was filtered into another round bottom flask. This process was repeated twice. After drying, a white film was observed on the inside of the round bottom flask (Figure 2).

DMSO-*d*_6_ was used as the NMR solvent to dissolve the entire TPA sample and a ^1^H-NMR was taken to confirm the presence of TPA as a depolymerization product. Bibenzyl was added as an internal standard and yield was calculated by NMR as follows:

Calculated the ratio of TPA (T) to bibenzyl (B):

(1) $$\frac{\frac{{\mathrm{integral}}_{T}}{{\mathrm{integral}}_{B}}}{\frac{{\mathrm{protons}}_{T}}{{\mathrm{protons}}_{B}}}={\mathrm{ratio}}_{T/B}$$

Calculated moles of bibenzyl:

(2) $$\frac{m_{B}}{{\mathrm{MW}}_{B}}={\mathrm{mol}}_{B}$$

Calculated moles of TPA:

(3) $${\mathrm{mol}}_{B}{\times \mathrm{ratio}}_{T/B}={\mathrm{mol}}_{T}$$

Calculated mass of TPA:

(4) $${\mathrm{mol}}_{T}{\times \mathrm{MW}}_{T}=m_{\mathrm{Texp}}$$

Calculated theoretical mass of TPA:

(5) $$m_{\mathrm{PET}}\times \frac{1}{{\mathrm{FW}}_{PET}}{\times \mathrm{MW}}_{T}=m_{\mathrm{Ttheo}}$$

Calculated experimental yield by NMR:

(6) $$\frac{m_{\mathrm{Texp}}}{m_{\mathrm{Ttheo}}}\times 100=\%\;\mathrm{yield}\;\mathrm{of}\;T$$

where m is mass, MW is molecular weight, FW is the formula weight.

The expected yield of terephthalic acid was calculated based on a theoretical 100% monomer consisting of one terephthalic acid and one ethylene glycol fragment. The reported yields are therefore likely an underestimation of the yield due to the prevalence of unknown additives and plasticizers [39]. When analyzed by GC-EI, evidence of potential plasticizers was found (discussed further in Section 4.5).

### 4.3. Microwave Experiments

Reactions were run in a 2450 MHz CEM Mars 6 Microwave (Matthews, NC, USA) equipped with a fiber optic temperature probe. 20 mL CEM GlassChem vessels equipped with magnetic stir bars were charged with a maximum of 14 mL of solvent per vessel and a minimum total solvent volume of 50 mL. Vessels were capped using standard GlassChem covers and a control vessel was equipped with a sapphire thermowell for temperature monitoring. All vessels were sealed to allow for pressure buildup. The reactions were ramped to temperature, maintaining a ramp rate from 11–13 °C and never exceeding 1000 W of power. 

### 4.4. Electrochemistry Experiments and Gas Chromatography

Controlled potential electrolyses for headspace analysis were performed in a custom H-cell equipped with the anode and cathode chambers separated by a glass frit with a separated headspace. The working electrode chamber contained a glassy carbon plate electrode and a BASi single junction Ag/AgCl (3 M NaCl) reference electrode. The counter electrode chamber contained a high-surface-area carbon cloth. Before electrolysis, the solution was sparged with argon and the cell was evacuated and backfilled with argon three times. Electrolyses were run with 20 mL in each chamber of a 50% MeOH in HPLC water solution with 0.1 M NaCl as supporting electrolyte. 

Gas chromatographic analysis was performed on a Hewlett Packard 5890 Series II gas chromatograph equipped with a thermal conductivity detector (TCD) and flame ionization detector (FID) in series with a Carboxen-1010 PLOT capillary column (Bellefonte, PA, USA). Argon was used as the carrier gas. The flow rate was 3 mL/min. Flow and make up was 6 mL/min. The reference gas flow was 19 mL/min. The FID has no auxiliary gas, air flow was at 350 mL/min and H_2_ was at 35 mL/min. The inlet was heated to 200 °C and the oven started at a temperature of 35 °C which was held for 8 min before a ramp of 20 °C/min up to 195 °C which was held for 1 min. A gas-tight analytical syringe (Hamilton 1750, 500 μL) was used to collect 200 μL aliquots for analysis. 

### 4.5. Identification of Plasticizers by GC-EI

Data were collected on a Thermo ISQ LT GC-MS in EI mode with an electron energy of 70 eV (Waltham, MA, USA). The column used was a Zebron ZB-5HT (30 m × 0.25 mm inside diameter, 0.25 μm film thickness, Newport Beach, CA, USA). Samples were collected in split mode with a column flow of 1 mL/min, purge flow of 10 mL/min, and a split flow of 10 mL/min. Inlet and ion source temperatures were held at 200 °C and 180 °C respectively. Samples for injection were prepared by diluting one drop of depolymerization NMR sample in 1 mL of methanol and an aliquot (1 μL) of the dilution was injected. The oven starting temperature was 70 °C held for 1 min followed by a ramp of 60 °C/min up to 300 °C which was held for 6 min. Data were collected using Chromeleon software. Mass spectra from peaks in the total ion chromatograph were compared to a NIST database using the software which listed possible matches with %probability. The results are summarized in Table 4.

## Figures and Tables

**Table 1:** Screening conditions for depolymerization of PET plastic.

Reaction Entry	Conditions ^a^	Reaction Time	Terephthalic Acid Yield% ^b^
**1**	MeOH, reflux	4 h	23
**2**	MeOH, reflux	12 h	75
**3**	Water, reflux	48 h	23

^a^ Reactions were run at reflux with 100 mg commercial PET plastic from a standard water bottle. ^b^ Yield based on terephthalic acid after acidic workup. Given the limitation of ether accelerants and advances in synthetic methodologies based on microwave reactors [29], combined with reports related to the recycling of PET plastics through microwave digestion in aqueous conditions with the use of phase-transfer catalysts [30], we proceeded to investigate the depolymerization of end-use PET in conditions similar to Table 1, Entry 2. In the microwave, temperatures above the solvent boiling points can be achieved, thus producing higher reaction rates and lowered reaction times. The results of our studies are summarized in Table 2.

**Table 2:** Microwave reactions for the depolymerization of poly(ethylene terephthalate).

Reaction Entry	Conditions ^a^	Reaction Time	Terephthalic Acid Yield%
**1**	Water, 170 °C	30 min	0
**2**	MeOH, 85 °C	13 min	55
**3**	MeOH, 85 °C	40 min	65
**4**	MeOH, 130 °C	40 min	65

^a^ Reactions were run in 20 mL CEM GlassChem vessels equipped with magnetic stir bars charged with 14 mL solvent and 10 mg end-use PET and 3.75 M NaOH with a maximum power of 1000 W.

**Table 3:** Reactions at room temperature (21 °C), for 1h in a batch electrolysis H-Cell divided by a coarse ceramic frit at controlled potential against a single-junction Ag/AgCl reference electrode with 20 mg PET plastic.

Reaction Entry	Conditions	Terephthalic Acid Yield% ^a^	nCO_2_ * 10^−6 b^
**1**	50% MeOH in water, −2.2 V	16.9	7.89 ± 0.36
**2**	Water, 0.1 M NaCl, −2.2 V	0.51	n/a ^c^

^a^ Yield of terephthalic acid obtained after the acidic workup of the cell contents. ^b^ Amount of CO_2_ produced in the anode was calculated from the quantitation of a CO_2_ sample from the anode headspace by GC-TCD (thermal conductivity detection) on a PLOT 1010 column quantified against standards. ^c^ CO_2_ signal present, but below quantitation limit.

**Table 4:** Potential plasticizers identified using GC-EI.

Compound	Prob (%)
Dodecamethylcyclohexasiloxane	86.89
Tetradecamethylcyclohexasiloxane	86.89
Hexadecamethylcyclooctasiloxane	75.73
Octasiloxane	66.82
